# Mueller Matrix Microscopy for In Vivo Scar Tissue Diagnostics and Treatment Evaluation

**DOI:** 10.3390/s22239349

**Published:** 2022-12-01

**Authors:** Lennart Jütte, Bernhard Roth

**Affiliations:** 1 Hannover Centre for Optical Technologies, Leibniz University Hannover, Nienburger Straße 17, 30167 Hannover, Germany; 2 Cluster of Excellence PhoenixD (Photonics, Optics and Engineering—Innovation Across Disciplines), Welfengarten 1A, 30167 Hannover, Germany

**Keywords:** polarimetry, dermoscopy, scar tissue, skin

## Abstract

Scars usually do not show strong contrast under standard skin examination relying on dermoscopes. They usually develop after skin injury when the body repairs the damaged tissue. In general, scars cause multiple types of distress such as movement restrictions, pain, itchiness and the psychological impact of the associated cosmetic disfigurement with no universally successful treatment option available at the moment. Scar treatment has significant economic impact as well. Mueller matrix polarimetry with integrated autofocus and automatic data registration can potentially improve scar assessment by the dermatologist and help to make the evaluation of the treatment outcome objective. Polarimetry can provide new physical parameters for an objective treatment evaluation. We show that Mueller matrix polarimetry can enable strong contrast for in vivo scar imaging. Additionally, our results indicate that the polarization stain images obtained form there could be a useful tool for dermatology. Furthermore, we demonstrate that polarimetry can be used to monitor wound healing, which may help prevent scarring altogether.

## 1. Introduction

Scars develop after skin injury when the body repairs the damaged collagen network. The treatment of scars amounts to about USD 20 billion per year in the US alone [1]. The standard device for human skin examination, the dermoscope, helps the dermatologist in visualizing subcutaneous structures that are not visible by the naked eye [2]. Only certain types of scars can be distinguished with the dermoscope [3]. However, the dermoscope or visual inspection without additional tools does not provide sufficient contrast to evaluate the treatment outcome with biomedical imaging techniques. This is particularly important as none of the currently available treatment options is universally successful [1]. As patients suffer from the psychological impact of a scar [4], their subjective assessment of a treatment cannot be trusted either. However, current scar assessment methods such as the Patient and Observer Scar Assessment Scale (POSAS) partially rely on patient scores in categories such as colour, pliability, thickness and relief, as well as itching and pain [5]. Therefore, a non-invasive and objective evaluation of the treatment outcome is necessary.

Dermatologists differentiate three types of scar tissue: keloids, hypertrophic scars and contracture scars [6]. Keloids appear about three months after injury, expand beyond the initial injury borders and do not regress over time. In contrast, the more common hypertrophic scars fade over time. Contracture scars usually build up after a burn injury and impair the movement of the skin area [7]. There are particular types of cancers that resemble one of these scar types. It is important to differentiate these malignancies from keloids or hypertrophic scars before treatment as, e.g., steroids can be contraindicated in malignant tumors [8].

Mueller matrix polarimetry (MMP) has a wide variety of possible applications in material sciences [9] and biomedical sciences [10], such as ophthalmology [11] and oncology [12]. With Mueller matrix polarimetry, it is possible to obtain information about the tissue composition and structure [13]. MMP has already been applied to numerous examples in skin research [14]. Quantitative measurements of the stratum corneum’s water content have been made for animal models [15], along with microstructures of skin hair follicles [16], the ability to monitor the microstructural changes of skin tissues during UVR-induced photo-damaging [17], the ex vivo detection of skin cancer [18] and optical parameters for calfskin [19]. Moreover, different types of skin cancer have been distinguished in human skin, with malignant melanoma displaying a higher degree of polarization (DOP) [20]. Additionally, attempts have been made to assess skin lesions [21] and detect melanoma with the help of machine learning [22]. Furthermore, the epidermis’ roughness has been assessed [23].

In this work we demonstrate fast and precise examination of skin tissue scars with an in-house MMP setup that provides spatially resolved Mueller matrices in relatively short time. We show that the procedure delivers useful information on the type and evolution of scar tissue, which can be beneficial to skin diagnostics in the future. In the next steps, we intend to apply the setup for systematic measurements in a clinical environment.

## 2. Materials and Methods

### 2.1. Mueller Formalism

Mueller matrix (MM) polarimetry can provide information about a sample’s polarization-changing characteristics within a 4 × 4 matrix [11]. Lights with different polarization states are used to illuminate the sample. The intensity of the transmitted or reflected light can be used to compute the MM [24]. The MM is a Stokes vector transformation matrix, which mathematically describes the polarization state of light. The Stokes vector of the incident light $S_{i}$ and the sample’s MM can be used to calculate the polarization state of the light following its interaction with the sample $S_{0}$ by [25]:

(1) $$\left(\begin{array}{c} S_{o1}\\ \begin{array}{c} S_{o2}\\ S_{o3}\\ S_{o4} \end{array} \end{array}\right)=\left[\begin{array}{cc} \begin{array}{cc} M_{11} & M_{12}\\ M_{21} & M_{22} \end{array} & \begin{array}{cc} M_{13} & M_{14}\\ M_{23} & M_{24} \end{array}\\ \begin{array}{cc} M_{31} & M_{32}\\ M_{41} & M_{42} \end{array} & \begin{array}{cc} M_{33} & M_{34}\\ M_{43} & M_{44} \end{array} \end{array}\right]\left(\begin{array}{c} S_{i1}\\ \begin{array}{c} S_{i2}\\ S_{i3}\\ S_{i4} \end{array} \end{array}\right)$$

Stokes vectors can be used to characterize these polarization states as follows [26]:

(2) $${\vec{S}}_{Stokes}=\left(\begin{array}{c} I_{H}+I_{V}\\ \begin{array}{c} I_{H}-I_{V}\\ I_{P}-I_{M}\\ I_{R}-I_{L} \end{array} \end{array}\right)=\left(\begin{array}{c} I_{H}+I_{V}\\ \begin{array}{c} I_{H}-I_{V}\\ 2I_{P}-(I_{H}+I_{V})\\ 2I_{R}-(I_{H}+I_{V}) \end{array} \end{array}\right)$$

The indices of the intensity values I, as indicated in Table 1, define the different polarization states of light required for polarimetry.

To determine the MM, the variations in the Stokes vector following the interaction of the incident light with the sample are recorded. For the used scenario, 36 (needed polarization states of the light: H, V, P, M, R, L) different measurements were generated [27]. In-depth explanations of the formalism can be found in the literature [25]. As the acquisition of the MM through 6 polarization states and the required 36 intensity measurements increase measurement accuracy by balancing calibration and measurement errors [28], we use this setting in this work. By taking measurements on samples of well known MMs such as polarizer and retarder samples as well as air, the polarimeter’s degree of calibration is evaluated. The literature provides a full description of the calibration procedures [29]. The findings demonstrate a very high degree of agreement with the expected matrices. The quantitative calibration results can be found in [27]. Particularly in the case of in vivo measurements, when motion blur can affect the result, the increase in measurement times requires careful attention.

The experimentally obtained MM entries do not exhibit a direct relationship to the sample’s physical characteristics. Instead, a polar decomposition is typically carried out for further measurement interpretation. In this study, we applied a spatially resolved version of the well known polar decomposition by Lu and Chipman [30]. The pixel array has an index of *i* for the rows and *j* for the columns.

(3) $$M_{exp,\;ij}=M_{\Delta ,ij}\cdot M_{R,ij}\cdot M_{D,ij}$$

In this decomposition, $M_{exp,ij}$ is the MM that was obtained experimentally, while $M_{\Delta ,ij}$*,* $M_{R,ij}$ and $M_{D,ij}$*,* respectively, reflect pure depolarizer, retarder and diattenuator behaviour [30].

The depolarization power Δ, total retardance *R*, diattenuation *D* and total polarizance *P* are the important metrics that partly result from the polar decomposition. When an incident beam interacts with a sample and the degree of polarization is reduced, the depolarization is increased. Different changes in the optical phase are produced when light travels through a birefringent medium for the ordinary and extraordinary polarization components. Retardance is the term for the variation in those phase shifts. When the polarization of the input beam is changed while the total intensity is maintained, the output intensity can vary. This is referred to as diattenuation [31]. The following equations describe the calculation of these physical properties of the sample [30]:

(4) $$\Delta =1-\frac{\left|M_{22}\right|+\left|M_{33}\right|+\left|M_{44}\right|}{3}$$

(5) $$R=cos^{-1}\left(\frac{tr\left(M_{R}\right)}{2}-1\right)$$

(6) $$P=\frac{1}{M_{11}}\sqrt{M_{21}^{2}+M_{31}^{2}+M_{41}^{2}}$$

(7) $$D=\frac{1}{M_{11}}\sqrt{M_{12}^{2}+M_{13}^{2}+M_{14}^{2}}$$

### 2.2. Polarization Staining

We visualize our results in such a way that they resemble dermoscopic images. Therefore, they can be intuitively interpreted by dermatologists. The agreement with the clinical practice could increase the acceptance by the dermatologists in addition. A visualization method of the polarimetric parameters derived from the Lu–Chipman decomposition is the polarization stain image (PSI). The PSI has been shown to help to identify locations of collagen, muscle fibers and connective tissue in skin tissue sections [32]. The method employed in this paper is based on the RGB colour space. The colour channels are filled with the spatially resolved polarization parameters according to Table 2.

**Figure 1:**
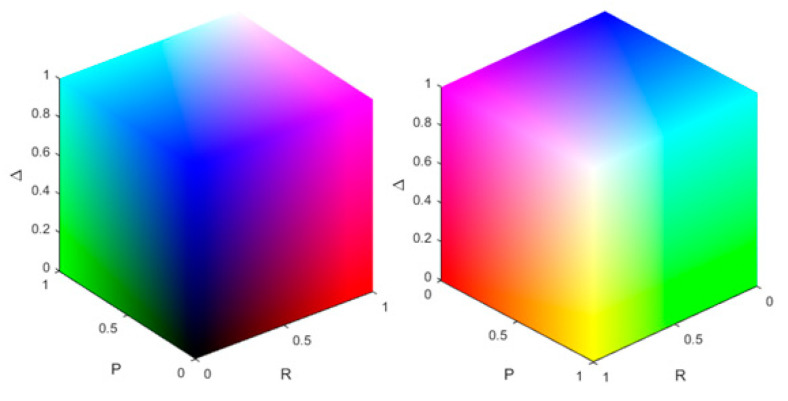
Visualization of colour scheme for all combinations of the intensity of the polarization parameters. The colour channels represent the physical properties obtained through the polar decomposition. The left and right image show the same colour cube from different perspectives.

The resulting colour scheme for the different combinations of the polarization parameters is shown in Figure 1. Whether or not a pixel is stained is decided by a threshold. Here, if the value of the polarization parameter is bigger than the mean of all the pixels of the same polarization parameter, we assign the pixel value of 255 to the related colour channel. In the remainder of this work, this method will be referred to as threshold staining. Another method for the PSI is to scale the range of the polarization parameter (e.g., 0 to 1) from its physical range to the range of the RGB colour space (0 to 255) which will be called range adjusted staining.

Further classical image processing techniques are applied to the PSI, i.e., noise reduction via Gaussian and median filtering. The speckle-noise that laser-based imaging can produce is considerably reduced by these image processing techniques and thus increases contrast [33].

### 2.3. Mueller Matrix Polarimeter

**Figure 2:**
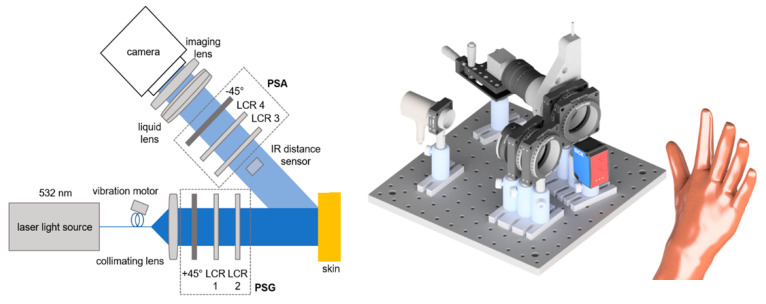
Sketch of Mueller matrix polarimeter. Left: 2D top view. Right: 3D side view rendering. The IR distance sensor is positioned below the beam path, not within it. The setup consists of the 532 nm continuous wave laser light source (CW532-04-1, ROITHNER LASERTECHNIK GmbH, Wien, Austria), liquid crystal retarder (LCC1223T, Thorlabs, Newton, NJ, USA), IR distance sensor (DT35-B15851, Sick AG, Waldkirch, Germany), liquid lens (EL-16-40-TC, Optotune AG, Dietikon, Schweiz) and CCD camera (BFS-U3-32S4M-C, FLIR Integrated Imaging Solutions Inc., Richmond, BC, Canada).

For the measurements detailed in this paper, we employ our in-house Mueller matrix polarimeter consisting of a laser light source, linear polarizers (+45°/−45°) and liquid crystal retarders (LCRs) as main elements, as depicted in Figure 2 [34].

To prevent crosstalk from the infrared laser, the distance sensor measures the distance at a location outside the polarimeter’s field of view (FoV) yet close to it. With the liquid lens, the focus can be changed in real time in accordance with the distance data provided by the sensor. Over a theoretical working distance of 30 cm to up to 8 m [27], the autofocus performs well for most of the skin parts. However, the targeted resolution and light intensity both have a limit on the operating distance. The acquisition time for one Mueller matrix depends on the exposure time but averages between 20 and 30 s. The distance from the camera to the skin is about 30 cm. In this scenario, the FoV is approximately 3 by 2 cm.

With the autofocus, it is possible to fully open the imaging lens’s aperture and accept a shallower depth of field because larger apertures reduce the acquisition time due to shorter necessary exposure durations. The reduced exposure times help to avoid motion blur as well.

## 3. Results

### 3.1. Scar Imaging

**Figure 3:**
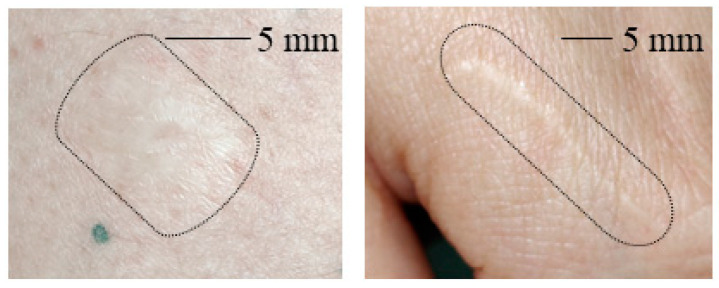
(**Left**): Photo of a contracture scar resulting from a burn. (**Right**): Photo of a hypertrophic scar. The scarred area on the skin is marked in each image.

Figure 3 shows clinical images of the scars studied in this work. The scarred skin area is marked by a black boundary in each image as the contrast to the healthy skin area is not strong enough to determine the scarred skin area directly.

From Figure 3 it is obvious that for the hypertrophic scar as well as for the contracture scar the scarred skin does not allow for strong contrast from healthy skin in clinical images. The scarred skin appears brighter but the contours are not clearly distinguishable in general.

All the measurements shown in the remainder of this work were obtained with a 1 mW diode laser and a laser with a wavelength of 532 nm (CW532-04-1, ROITHNER LASERTECHNIK GmbH, Wien, Austria) with our in-house polarimetry system adapted for this study with regard to working distance, measurement duration and FoV.

**Figure 4:**
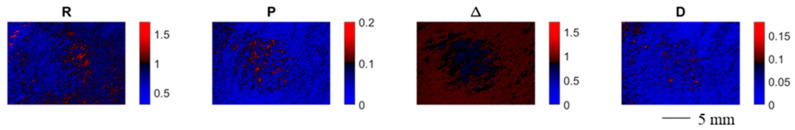
Spatially resolved polarimetric parameters for an exemplary contracture scar. Each polarimetric parameter provides a certain degree of contrast between the scarred and the healthy skin area.

Figure 4 shows the spatially resolved polarimetric parameters for an exemplary contracture scar. Each polarimetric parameter provides a certain degree of contrast between the scarred and the healthy skin area. The depolarization power Δ provides the strongest contrast for scarred and healthy skin, which could be due to a higher degree of orientation of the collagen fibers for the scarred tissue hypothesized in the literature [35].

**Figure 5:**
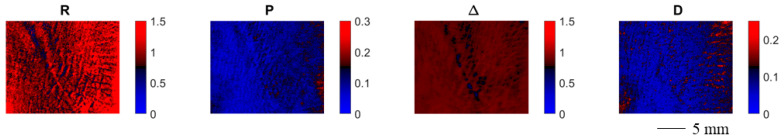
Spatially resolved polarimetric parameters for an exemplary hypertrophic scar. Each polarimetric parameter provides a certain degree of contrast between the scarred and the healthy skin area.

In Figure 5, we display the spatially resolved polarimetric parameters for an exemplary hypertrophic scar. The contrast between the damaged and the healthy skin patches varies depending on the polarimetric parameter. The depolarization power allows for the clearest display of the scarred skin areas.

**Figure 6:**
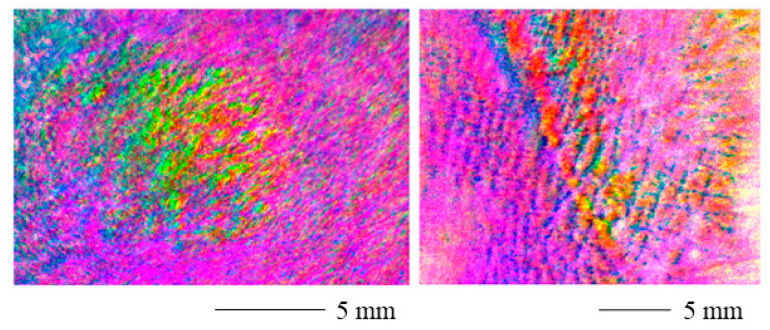
Range adjusted polarization stain image for a contracture scar (**left**) and a hypertrophic scar (**right**). The scarred areas are clearly distinguishable. The values shift toward the edges of the image and the curved skin geometry may be the cause of this.

The range adjusted polarization stain images are displayed in Figure 6 for a contracture scar (left) and a hypertrophic scar (right). The scarred areas are easily distinguished from the healthy skin. However, the values shift toward the edges of the image. The reason for this might be the curved skin geometry.

**Figure 7:**
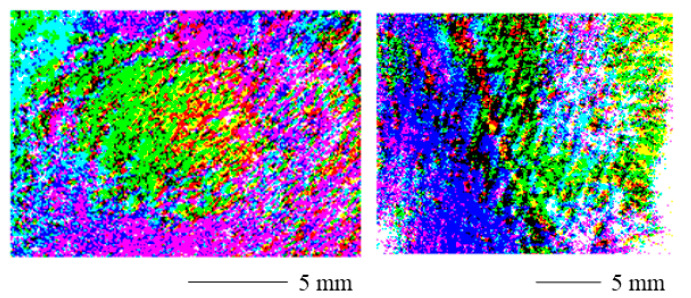
Threshold polarization stain image for a contracture scar (**left**) and a hypertrophic scar (**right**). The scarred portions can easily be identified. Near the margins of the image, the values change. The curved skin geometry could be the cause of this.

Figure 7 displays the threshold polarization stain images for a contracture scar (left) and a hypertrophic scar (right). The scarred skin can easily be identified. Near the margins of the image, the values change as for the range adjusted PSI. Again, the curved skin geometry could be the cause of this as the degree of polarization can be affected by the surface normal [36] and therefore also the surface roughness [37,38] of the skin. Therefore, results from complex skin topographies and roughness might be difficult to interpret. Additionally, the signal-to-noise ratio could increase near the edges of the field of view in the case of non-uniform illumination [39].

### 3.2. Wound Healing Monitoring

**Figure 8:**
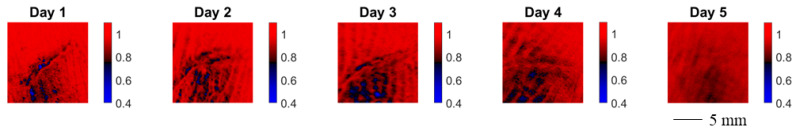
Spatially resolved depolarization power Δ for wound healing monitoring of a cut in a finger. The wound was measured five consecutive days after the cut. The orientation of the wound in the image changes in between measurements.

In the following, we study the wound healing process of an accidental cut in a finger with a knife. The healing process was documented by means of MMP on five consecutive days starting with the day after the injury. In Figure 8 we show the evolution of the spatially resolved depolarization power Δ. Note that the orientation of the wound in the image changes in between measurements.

Over the course of the measurement time, the spatially resolved depolarization power, as shown in Figure 8, becomes more homogeneous. The contrast of the injured skin and the healthy skin vanishes.

A similar result is obtained for the spatially resolved diattenuation wound healing monitoring.

**Figure 9:**
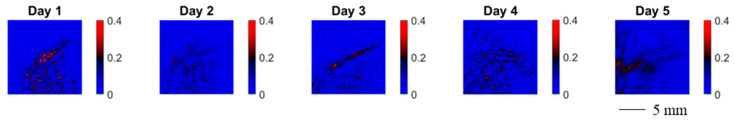
Spatially resolved diattenuation D for wound healing monitoring of a cut in a finger. The wound was measured five consecutive days after the cut. Between measurements, the wound’s orientation changes in the image.

**Figure 10:**
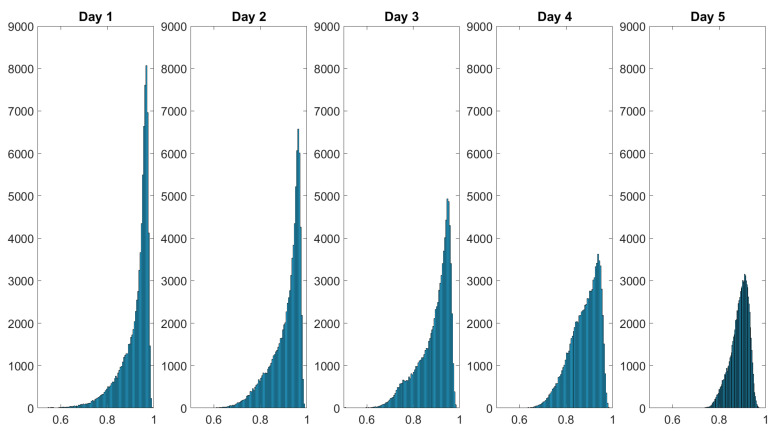
Histograms of the depolarization power Δ data monitoring the wound healing of a finger cut. The intensity distribution of Δ flattens over the healing time. The dataset’s values are shown on the *x*-axis and the frequency of each value is shown on the *y*-axis. The values in the dataset are displayed on the *x*-axis and the frequency of each value is displayed on the *y*-axis.

The spatially resolved diattenuation shown in Figure 9 seems to become more homogenous as well. The histograms of the results displayed in Figure 8 and Figure 9 are examined to further explore this phenomenon. The histograms visualize the statistical distribution of the acquired data. We ensured that the number of pixels imaging the skin area under study is constant by cropping the spatially resolved polarimetric parameters to the same size. We thus ensure that the injury occupies the same amount of the FoV because the data were collected from a constant working distance. The histograms of the depolarization power Δ data monitoring the wound healing of a finger cut are displayed in Figure 10.

**Figure 11:**
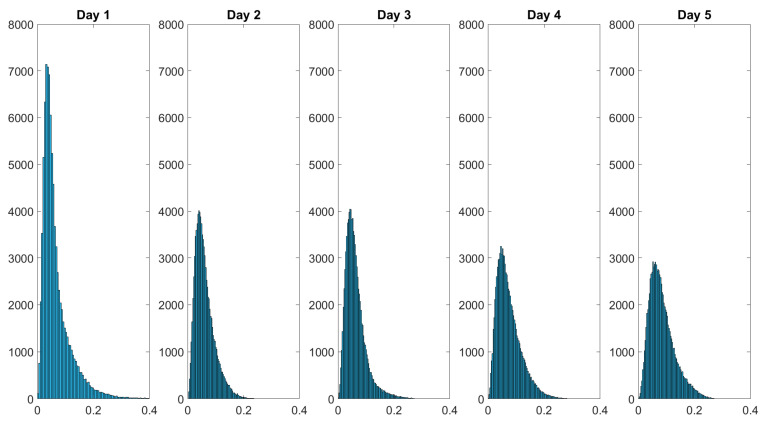
Histograms of the diattenuation D data monitoring the wound healing of a finger cut. The intensity distribution of D flattens over the healing time. The values in the dataset are displayed on the *x*-axis and the frequency of each value is displayed on the *y*-axis.

Over the course of the measurement period, the histograms of the spatially resolved depolarization power flatten while also displaying broadening. The initial negative skewed distribution changes throughout the course of the measurement period into a more bell-shaped distribution at day 5. Figure 11 shows the histogram of the diattenuation for the wound healing of a finger cut.

The statistical distribution of the diattenuation changes from positive skewed to slightly bell-shaped within the five measurement days. In the following, we study the distributions with the Pearson coefficient and the kurtosis. The Pearson coefficient describes the skewness, while the kurtosis is related to the degree of peakness of a statistical distribution. These characteristics may be more useful for analyzing the histograms than the mean and median values alone because the skin composition of healthy skin may differ for various body areas.

As can be seen in Table 3, the Pearson coefficients for the depolarization power and the diattenuation tend towards zero, which would describe the symmetric distribution.

The histograms presented in Figure 10 and Figure 11 as well as the Pearson coefficients and kurtosis values of the statistical data distributions shown in Table 3 indicate that MMP is a suitable tool for non-invasive in vivo wound healing monitoring in human skin.

## 4. Conclusions

In this work, we report on the application of in vivo MMP for objective scar assessment beyond the subjective state-of-the-art scar assessment methods with scores for scar colour, pliability, thickness, relief, itching, pain, vascularization, pigmentation and a general rating of appearance as for the POSAS [5].

Non-invasive methods for in vivo scar assessment are necessary as the treatment of scars has a huge economic impact and the cosmetic disfigurement of scarring also has negative psychological effects for each patient. As no treatment is universally successful, an objective evaluation of the scar treatment is needed. We show that MMP could be a promising tool to provide objective measures for the assessment of scar tissue. In addition, it is of great importance to easily distinguish scar tissue from possible malignancies. As the melanoma incidence is rising there will be more excisions possibly resulting in scarring. The main findings of this work are the strong contrast of scar tissue and healthy skin through polarimetric microscopy, the usability of the polarization stain image for possible applications in dermatology and the possibility of wound healing monitoring by the means of MMP.

The developed method based on polarimetry will be tested within a clinical context in the next step. In this context, certain stages of scarring can be evaluated. Moreover, wound healing processes for different types of wounds and the corresponding scar development as well as the effect of medical interventions (i.e., topical, intralesional, cryo, laser or mechanical therapy [1]) could be monitored. Further, a stronger connection between the medical information and the polarimetric parameters needs to be drawn. There are several diseases in which the MMP imaging could be of dermatologic value, e.g., the inflammatory skin disease morphea, which causes a hardening of the skin. Here, the distribution of collagen plays an important role. For this purpose, combination of the technique with other optical imaging modalities or optical clearing for greater penetration depths [40] might be beneficial and will be explored as well [41,42,43]. Beyond that, collagen is important in scar development, for example, in wound healing or acne. Further work might include the testing of distinguishability of keloids and hypertrophic or contracture scars.

## Figures and Tables

**Table 1:** Index and polarization state correlation.

**Index**	H	V	P	M	R	L
**Polarization**	horizontal	vertical	linear +45°	linear −45°	right circular	left circular

**Table 2:** Polarization staining in RGB colour space. Each colour channel is related to a physical property.

**Colour channel**	R	G	B
**Polarization parameter**	Retardance	Polarizance	Depolarization Power

**Table 3:** Pearson coefficients and kurtosis values of the statistical data distributions of the wound healing monitoring.

Day		1	2	3	4	5
Δ	Pearson coefficient	−1.1469	−1.0464	−0.9031	−0.4783	−0.5967
	Kurtosis	20.6685	4.4356	4.1774	2.9353	3.3881
D	Pearson coefficient	0.9390	0.6858	0.6586	0.6572	0.5807
	Kurtosis	14.0680	5.4770	8.0739	4.7647	4.2798

## Data Availability

The data that support the findings of this study are available from the corresponding author upon reasonable request.
